# An atlas of tsetse and bovine trypanosomosis in Sudan

**DOI:** 10.1186/s13071-016-1485-6

**Published:** 2016-04-07

**Authors:** Selma K. Ahmed, Ahmed H. Rahman, Mohammed A. Hassan, Sir Elkhatim M. Salih, Massimo Paone, Giuliano Cecchi

**Affiliations:** Veterinary Research Institute, Khartoum, Sudan; Veterinary Research Institute, Damazine Regional Laboratory, Damazine, Sudan; Food and Agriculture Organization of the United Nations, Animal Production and Health Division, Rome, Italy; Food and Agriculture Organization of the United Nations, Sub-Regional Office for Eastern Africa, Addis Ababa, Ethiopia

**Keywords:** Tsetse, *Glossina*, African animal trypanosomosis, GIS, Atlas, Sudan, Epidemiology

## Abstract

**Background:**

After a long period of neglect, initiatives are being implemented in Sudan to control tsetse and trypanosomosis. Their planning, execution and monitoring require reliable information on the geographic distribution of the disease and its vectors. However, geo-referenced and harmonized data at the national level are lacking, despite the fact that a number of epidemiological studies were conducted over the years. The Atlas of tsetse and bovine trypanosomosis in Sudan tries to fill this gap.

**Methods:**

The present study includes both a review of historical datasets on tsetse flies and bovine trypanosomosis, as well as the results of recent, targeted field investigations. The review includes both published and unpublished datasets collected in Sudan from 1960 onwards. Targeted field investigations were conducted for trypanosomosis in Blue Nile (2011) and Gezeira States (2012), for tsetse flies in South Darfur (2012) and Blue Nile States (2009 and 2011), and for other trypanosomosis vectors in seven States (Khartoum, Gezeira, White Nile, Blue Nile, North Kordofan, Kassala and Gadarif). The latter surveys, conducted from 2010 to 2012, also enabled us to confirm the absence of tsetse flies in a number of locations.

**Results:**

Tsetse fly infestation in Sudan appears to be limited to two relatively small areas at the south-western and south-eastern tips of the Country (South Darfur and Blue Nile State respectively)*. Glossina morsitans submorsitans* is present in both areas, whilst *G. fuscipes fuscipes* is found only in the latter. In contrast, bovine trypanosomosis is widespread, its presence having being confirmed in eleven States and suspected in all the others. Both mechanical transmission by non-cyclical vectors and animal movement contribute to this broad distribution of trypanosomosis. This is especially the case for *Trypanosoma vivax*, which was found even in sedentary cattle at hundreds of kilometres of the tsetse belt.

**Conclusions:**

The Atlas provides a spatially-explicit synthesis of the current knowledge of tsetse and bovine trypanosomosis in Sudan. Its various epidemiological outputs are being used to target both trypanosomosis control activities and further data collection exercises. Activities are ongoing to expand the Atlas to non-cyclical vectors and hosts other than cattle.

**Electronic supplementary material:**

The online version of this article (doi:10.1186/s13071-016-1485-6) contains supplementary material, which is available to authorized users.

## Background

Trypanosomes are flagellate protozoans that inhabit the blood plasma, the lymph and various tissues of their hosts. They are the aetiological agents of a group of parasitic diseases (trypanosomoses) that cause serious economic losses and impose a severe public health burden [1–3].

In Sudan, African animal trypanosomosis (AAT) is present in the tsetse-infested areas and beyond. Mechanical transmission by other biting flies and animal movement contribute to spreading the disease in tsetse-free areas. By contrast, following its partition from South Sudan, the country is not considered endemic for sleeping sickness, the human form of the disease [4–6].

Bovine trypanosomosis is one of the most economically important diseases affecting domesticated animals in Sudan, a country where agro-pastoralism is the dominant system of production [7]. In one of the few economic studies conducted in the country (Blue Nile State), trypanosomosis was reported as the cause of high calf mortality (> 70 %), high abortion rates (> 70 %), as well as a reduction in milk production (25 %) [8]. In the same study, the costs of treating the disease, an indirect economic impact of trypanosomosis, were estimated to exceed one million Sudanese pounds per year (equivalent to 300,000 USD) in the Blue Nile State only. The National Medicines and Poisons Board of Sudan reported that imports of trypanocidal drugs at the national level cost over 28 million Sudanese pounds a year (over 11 million USD) [8]. Also, in a recent economic study for Eastern Africa, it was estimated that removing bovine trypanosomosis from tsetse-infested areas of Sudan would bring benefits of up to 12,500 USD per km^2^ over a 20-year period [2].

The first available record of the presence of AAT in Sudan appeared in 1904 in the annual report of the veterinary department [9]. The main pathogenic trypanosome species encountered in the Country are *Trypanosoma vivax, T. congolense, T. brucei, T. simiae* and *T. evansi*, with the first three being the most widespread in cattle. In previous studies reporting the presence of tsetse species, before separation from South Sudan, seven were described in the country, i.e. *Glossina morsitans submorsitans, G. tachinoides, G. pallidipes, G. fuscipes fuscipes, G. longipennis, G. fuscipleuris* and *G. fusca* [10]. After separation, only two species are reported as remaining on Sudanese territory: *G. morsitans submorsitans* and *G. fuscipes fuscipes.*

Since the successful eradication of *G. morsitans submorsitans* in 1961–1962 from Koalib Hills in South Kordofan [11], tsetse control was neglected in Sudan for many decades. Some impetus was only recently regained, also thanks to the advocacy action of the Pan African Tsetse and Trypanosomosis Eradication Campaign (PATTEC), an initiative of the African Union. In 2011, the Government of Sudan launched a trypanosomosis control project in South Darfur (south-western Sudan), which relies on pour-on formulations for the control of the disease vectors (i.e. tsetse and other biting flies). Economic models indicate insecticide-treated cattle as being the most cost-effective tsetse control technique where a sufficient number of bovines is available [12, 13]. With some training support from development partners, similar control activities were planned in the Blue Nile State (south-eastern Sudan).

These recent as well as forthcoming control initiatives require reliable information on the geographic distribution of AAT and of its vectors in Sudan. Despite the fact that various epidemiological studies were conducted over the years, geo-referenced and harmonized data on tsetse and AAT occurrence at the national level are lacking. This type of information is crucial for evidence-based planning, execution and monitoring of field interventions.

It is to fill this gap that the Veterinary Research Institute of Sudan (VRI) embarked on a national level mapping exercise, aimed at building a geo-referenced database of tsetse and bovine trypanosomosis for Sudan. The present paper reports on the results of this study. The initiative is technically supported by the Food and Agriculture Organization of the United Nations (FAO), in particular in the framework of the Atlas of tsetse and AAT [14, 15]. The continental Atlas of tsetse and AAT is an initiative of FAO, jointly implemented with the International Atomic Energy Agency (IAEA) in the framework of the Programme Against African Trypanosomosis (PAAT).

## Methods

The present study includes both a review of pre-existing datasets on tsetse and bovine trypanosomosis in Sudan, as well as the results of recent, targeted field investigations.

### Review of pre-existing datasets

The review on tsetse and bovine trypanosomosis in Sudan relied on methods developed by FAO for the continental Atlas of tsetse and AAT [14, 15]. In a nutshell, all publications and input data obtained from a variety of sources were collated and stored in a centralized data repository. Subsequently, selected pieces of information were extracted, harmonized and entered into a geo-spatial database. The database is structured into simple tables devoted to epidemiological and entomological data, geographical information and data sources.

As concerns the input datasets, the present study relied on published and unpublished sources, including peer-reviewed articles, PhD and MSc theses and reports by veterinary authorities at the national and sub-national level. With regard to AAT, the study focused on bovines, the livestock species in which trypanosomosis causes the heaviest economic losses [2]. All data from 1960 onwards were included.

Efforts were made accurately to map the available epidemiological data. A number of field missions were specifically carried out to geo-reference locations of interest by means of the Global Positioning System (GPS).

### Targeted field investigations

#### Tsetse flies

Two consecutive entomological surveys targeted at tsetse flies were conducted in the Blue Nile State in 2009 and 2011. A total of 30 biconical traps were deployed in 15 sites along the Yabus River for a duration of 8 days. Pre-existing information indicated that this area is infested by *G. fuscipes fuscipes* [16].

In addition to the targeted tsetse surveys in the Blue Nile State, a number of entomological surveys were also carried out between 2010 and 2012 in seven states (i.e. Khartoum, Gezeira, White Nile, Blue Nile, North Kordofan, Kassala and Gadarif) [17]. The vast majority of the surveyed areas were known to be free of tsetse, and therefore the investigations were mainly targeted at tabanids and other biting flies. Five Localities in each State were opportunistically sampled, in an effort to ensure fair coverage at the State level. Two NZI traps were deployed in each Locality. The NZI trap was developed as a tool to monitor both tsetse and biting flies [18]. For the present survey, traps were positioned in a range of ecotypes, including riverine vegetation, watering points, farmlands and grazing areas, and they were emptied after 72 h. As concerns tabanids and other biting flies, the results of these surveys will be presented elsewhere; in the present paper, only the absence of tsetse catches is discussed.

#### Bovine trypanosomosis

Targeted field investigations on bovine trypanosomosis were carried out in Blue Nile and Gezeira States to complement historical data.

With the support of FAO, the Blue Nile State was targeted by an AAT survey in April–May 2011. In this tsetse-infested state at the border with Ethiopia, transhumance contributes to a complex and dynamic epidemiological picture, with transboundary circulation of trypanosome strains at varying levels of virulence and occasional severe outbreaks [19]. Blood samples (302) were randomly collected from sedentary and transhumant cows in five Localities, i.e. Damazine (34), Elroseires (28), Baw (15), Quissan (85) and Kurmuk (125).

In November 2012, a similar survey was conducted in Gezeira State because of a recent AAT outbreak [20] in an area that had previously been considered unaffected by the disease [21]. A total of 100 blood samples were randomly collected from sedentary cows in four Localities (i.e. South Gezeira, Wad Madani, Elmanagil and East Gezeira). Forty samples were collected in South Gezeira, and 20 samples in each of the other three localities.

In both surveys (Blue Nile and Gezeira States), the presence of pathogenic trypanosomes was investigated with parasitological techniques. Five ml of blood were collected from the jugular vein of cows into heparinized vacutainers. The collected samples were examined with the Haematocrit Centrifugation Technique (HCT) [22], and species identification was carried out by means of Giemsa-stained thin smears.

## Results

As regards the review of historical data, a total of 54 sources were identified and processed, including scientific publications (20), PhD (4) and MSc (7) theses, and reports of veterinary authorities (23). The complete list of sources is provided in the Additional file 1. Targeted field investigations for bovine trypanosomosis and tsetse corroborated and updated the epidemiological picture that emerged from the review. Over 150 locations of epidemiological interest were geo-referenced and mapped. The combined results of the review and the targeted field investigations are presented below.

### Tsetse distribution

**Fig. 1:**
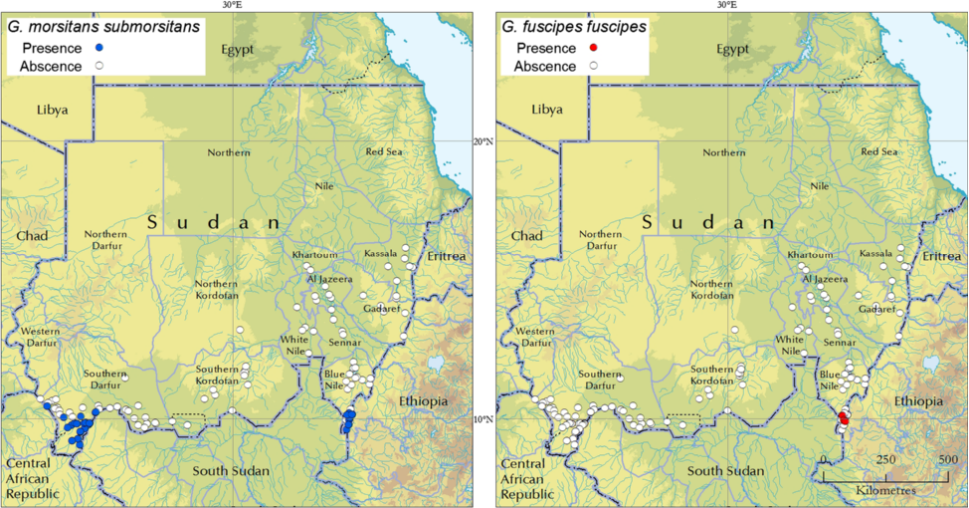
The distribution of *G. morsitans submorsitans* and *G. fuscipes fuscipes* in Sudan (1960-2012). For the Nuba Mountains area (South Kordofan) post-eradication absence of *G. morsitans submorsitans* is shown [11, 23]

The review of historical data indicated that tsetse fly infestation in Sudan is limited to two relatively small areas in the States of Blue Nile and South Darfur (Fig. 1). South Kordofan was historically infested, but no flies were ever detected in the State after the successful eradication of *G. morsitans submorsitans* from the Nuba Mountains in the early sixties [11, 23].

The occurrence of *G. fuscipes fuscipes* appears to be limited to the riverine habitats fringing the Yabus River at the southern tip of the Blue Nile State. Its distribution also extends into neighbouring Ethiopia [24]. The occurrence of *G. fuscipes fuscipes* in this area was confirmed by the field investigations conducted in 2009 and 2011. A total of 623 flies were caught, with an average apparent density of 2.6 flies/trap/day. In the Yabus District (Blue Nile State), *G. fuscipes fuscipes* coexists with *G. morsitans submorsitans*, which inhabits drier woodland areas.

*G. morsitans submorsitans* is also present in South Darfur, Radom Locality. In this area, relatively higher densities are found inside the Radom National Park.

The surveys conducted with NZI traps between 2010 and 2012, mainly targeted at tabanids, corroborated the notion that *Glossina* spp. are absent from the vast majority of the territory of Sudan [17]. The tsetse free area includes the surveyed States of Khartoum, Gezeira, White Nile, North Kordofan, Kassala and Gadarif. Although not surveyed, tsetse flies are also known to be absent from the Northern, Red Sea and North Darfur States, which are for the most part covered by deserts.

### Bovine trypanosomosis

**Fig. 2:**
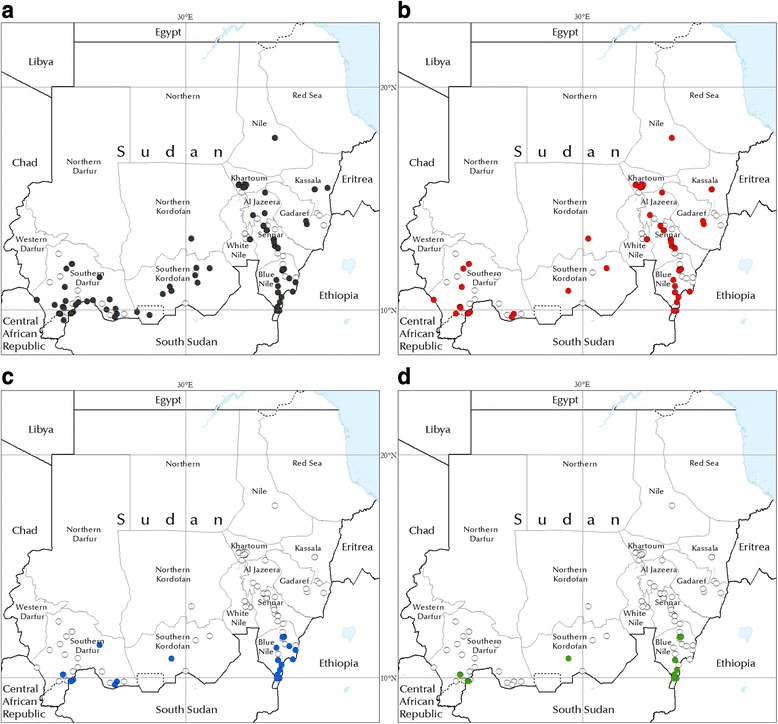
Absence of detection and presence of (**a**) trypanosomosis, (**b**) *T. vivax*, (**c**) *T. congolense*, and (**d**) *T. brucei* in bovines. White circles: absence of detection. Coloured circles: presence. Reporting period: January 1980 – December 2014. Diagnostic methods: smears, haematocrit centrifugation technique (HCT), buffy-coat technique (BCT), Polymerase Chain Reaction (PCR)

As compared to the very limited distribution of *Glossina* spp. (only present in relatively circumscribed areas of the Blue Nile and South Darfur States), bovine trypanosomosis is widespread in Sudan. The present study confirmed its presence in at least eleven States (Fig. 2). Data are lacking for three States in the northern part of the Country (Northern, Red Sea and North Darfur States).

*T. vivax* is by far the most widely distributed bovine-infective trypanosome species in Sudan. Because of its well described capability of also being transmitted mechanically [25, 26], its circulation is maintained by biting flies well beyond the tsetse belt. Cases of *T. vivax* were detected in resident cattle in the River Nile State, hundreds of kilometres from the tsetse-infested areas.

Cattle movement contributes to spreading the disease beyond the tsetse belt. As an example, in Khartoum State mechanical transmission and cattle trade linked to the presence of four large cattle markets have made *T. vivax* an endemic parasite. In a few farms in Khartoum state, prevalences of up to 100 % have been detected even in resident cattle [27]. In this context, the gathering of dairy farms with low biosecurity is facilitating the spread of the diseases.

**Fig. 3:**
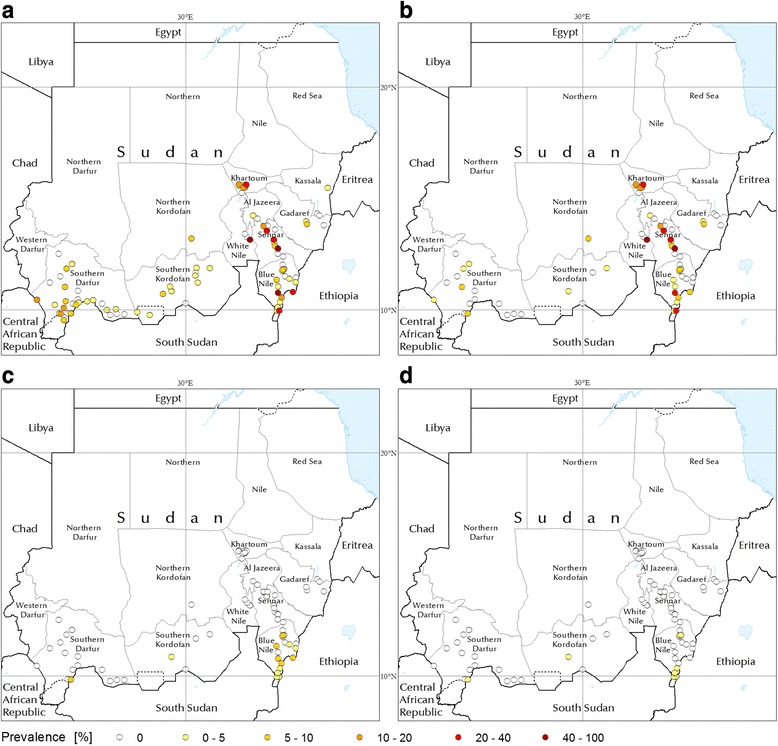
The prevalence of bovine trypanosomosis in Sudan (1980- 2014). **a** bovine trypanosomosis, (**b**) *T. vivax*, (**c**) *T congolense*, (**d**) *T. brucei*. Diagnostic methods: haematocrit centrifugation technique (HCT) and buffy-coat technique (BCT). Minimum sample size: 20 animals

As shown in Fig. 3, the highest *T. vivax* infection rates are to be found in the areas around the river Nile. The epidemiology of *T. vivax* in Sudan is fairly dynamic, with outbreaks that can be triggered by animal movement (e.g. trade or pastoralism). As an example, an outbreak occurred in the Gezeira State in 2007 [20], in a previously unaffected area [21]. Subsequent monitoring after extensive treatment of infected animals was the object of one of the targeted AAT investigations carried out for the present study. The results of the survey suggested that *T. vivax* had not persisted after the outbreak and the subsequent extensive treatment of infected animals, as none of the 100 sampled cows was found to be infected with trypanosomes. *T. vivax* was also found to be the dominant species in the survey of 302 cows carried out in an area of the Blue Nile State, in close proximity to the tsetse belt.

The distribution of *T. congolense* and *T. brucei* is more closely linked to the areas of *Glossina* infestation, although they can also be found at some distance from the tsetse belt. This is for example the case in the tsetse-free South Kordofan, where animal movement to and from tsetse-infested areas in South Sudan probably explains the occurrence of these two parasite species.

Where *Glossina* spp. are present, the prevalence of *T. congolense* and *T. brucei* can be on par with that of *T. vivax* (e.g. in Blue Nile State [19]) or even exceed it, as it is the case for *T. congolense* in *G. morsitans submorsitans*-infested South Darfur [28].

## Discussion

Obtaining a meaningful epidemiological picture of tsetse and bovine trypanosomosis in Sudan required the review of data collected over more than half a century, as well as targeted field investigations. In addition to the relative scarcity of data, methodological challenges included geo-referencing locations and varying levels of accuracy, variable and generally low sensitivity of the prevailing diagnostic tools, as well as other heterogeneities stemming from husbandry systems and the use of trypanocidal drugs.

Despite the still numerous gaps in space and time and the above heterogeneities, a fairly clear picture emerges: *Glossina* species are confined to very small areas while trypanosomosis, especially *T. vivax*, is widespread. A detailed discussion of the causes of this pattern is beyond the scope of the paper. Suffice it to say that mechanical transmission by biting flies and cattle movement are likely to be the main factors at play [29]. One of the consequences of this observation is that, in Sudan, bovine trypanosomosis in tsetse-free areas is so widespread that economic impacts based on the tsetse distribution alone (e.g. [2]) vastly underestimate the problem.

Although the review of published and unpublished data spanned a period of over 50 years, data gaps are still numerous. This can be ascribed to the lack of a national-level monitoring and reporting system for trypanosomosis, as well as the inefficiencies of the decentralized diagnostic facilities. As a result, most of the data included in the Atlas stem from research activities. Furthermore, trypanosomosis diagnosis in Sudan still relies to a great extent on low-sensitivity parasitological methods (e.g. HCT and BCT), while more accurate molecular tools (e.g. PCR) are rarely used. In the few instances where molecular tools were utilized, they predictably detected a higher prevalence than parasitological methods (e.g. in the Blue Nile State [19]). Also, PCR revealed more complex patterns of single as well as mixed infections, with up to four different trypanosome species, including *T. simiae* [19].

Looking at the trypanosomosis maps, there is an evident data gap in the north of the country (i.e. Northern, Red Sea and North Darfur States), where nonetheless the disease is suspected because of clinical reports as well as the presence of non-cyclical vectors. Future investigations in these areas could shift the limit of *T. vivax* distribution further north, beyond its 17°40′ N present latitude.

Not only is the problem of trypanosomosis in Sudan widespread, it also appears to be on the rise. Evidence from a few comparable epidemiological studies carried out at a distance of a few years suggests an increase of the prevalence as well as the geographical spread [30].

This apparent increase in the burden of trypanosomosis could be ascribed to a variety of interplaying factors, many of which are driving changes in disease landscapes at the global level [31]. In Sudan, the expansion of irrigated agriculture is often associated with an increased and intensified animal production, especially in dairy farms and fattening centres [30]. This process brings susceptible hosts and vectors closer together, especially tabanids. Lack of awareness of the disease and increased and uncontrolled long-range movement of animals, also from tsetse-infested areas, compound the problem. Intensification of animal production and close-herding seem to be particularly relevant, for example in the Khartoum State, as a large number of farms are increasingly brought together in very close proximity. Last but not least, limited investment in the strengthening of the veterinary services contributes to the problem.

The rising impact of trypanosomosis could also be exacerbated by drug resistance [32], in a context where chemotherapy is still by and large the cornerstone of trypanosomosis control in Sudan. To address this problem, in Khartoum State the Isometamidium chloride recently replaced Dimenazine as the main trypanosomosis control method [33].

## Conclusions

The Atlas of tsetse and bovine trypanosomosis in Sudan provides a range of epidemiological outputs that are being used to target both trypanosomosis control activities and further data collection exercises. In 2012, VRI initiated a few tsetse control trials against *G. morsitans submorsitans* in the Radom Locality, South Darfur State, in the framework of the PATTEC initiative, and activities are still ongoing. In the Blue Nile State, data collection exercises and pilot tsetse control trials were undertaken, with a view to carrying out similar control activities, but civil strife is presently preventing further implementation.

A number of activities will be undertaken to improve the Atlas. Surveys are being planned to fill the knowledge gaps (e.g. in the northern States), and work is ongoing to expand the Atlas to non-cyclical vectors (especially tabanids) and hosts other than cattle. Also, efforts will be made to scale up the use of more accurate diagnostic tools (e.g. PCR), whose availability to diagnose trypanosomosis in Sudan is still very limited. The Atlas of tsetse and bovine trypanosomosis in Sudan will also continue to contribute to, and benefit from, ongoing continental mapping initiatives [14, 15], and it could also provide input to modelling exercises [34, 35].

## Additional file

Additional file 1: List of sources analysed to generate distribution maps of tsetse and bovine trypanosomosis in Sudan. (DOCX 34 kb)
