# Supplementary material for: An atlas of tsetse and bovine trypanosomosis in Sudan
Source: Parasit Vectors. 2016 Apr 7;9:194. doi: 10.1186/s13071-016-1485-6 (PMC4825090; doi:10.1186/s13071-016-1485-6)
Supplement: Additional file 1: — List of sources analysed to generate distribution maps of tsetse and bovine trypanosomosis in Sudan. (DOCX 34 kb) [file 13071_2016_1485_MOESM1_ESM.docx]

**List of sources analysed to generate distribution maps of tsetse and bovine trypanosomosis in Sudan**

[1-54]

1. Abdalla M, Siham ES, Amel OB: ***Trypanosoma vivax* infection in Sudanese cattle in central Sudan**. *J Anim Vet Adv* 2005, **4**(11):945-948.

2. Abdalla M, Amel OB, Seham ES: **Seasonal prevalence ofbovine trypanosomosis in Abu Bugar District, Central Sudan**. *Sud J Vet Sci Anim Husb* 2008, **47**:1-5.

3. Abdel Razig M, Yagi A: **The distribution and advance of *Glossina morsitans* in southern Darfur District, Sudan**. *Bull Epizoot Dis Afr* 1973, **21**(3):253-258.

4. Adam ME: **Prevalence of blood parasites in domestic animals in south Darfur state of Sudan**. *MSc thesis.* Khartoum: University of Khartoum; 2005.

5. Gumaa M, Abusalab S, Omer M, Salih D, Mulla S, Omer E, Ahmed A: **A two year study on bovine trypanosomosis in Kassala State, Eastern Sudan (2007-2008)**. *Int Res J Agric Sci Soil Sci* 2011, **1**(3):96-97.

6. Hall M, Kheir S, Rahman A, Noga S: **Tsetse and trypanosomiasis survey of southern darfur province, Sudan. I. Bovine trypanosomiasis**. *Trop Anim Health Pro* 1983, **15**(4):191-206.

7. Hall M, Kheir S, Rahman A, Noga S: **Tsetse and trypanosomiasis survey of southern darfur province, Sudan. II. Entomological aspects**. *Trop Anim Health Pro* 1984, **16**(3):127-140.

8. Hassan MA: **Epidemiology of Trypanosomal infections in cattle in South Darfur state**. *MSc thesis.* Khartoum: University of Khartoum; 2003.

9. Hassan MA: **Evaluation of different tsetse intervention methods in the control of *Glossina fuscipes fuscipes* in Yabus area, Blue Nile state**. *PhD thesis.* Khartoum: Sudan Academy of Sciences; In preparation.

10. Husna IB: **The use of Antibody detection Enzyme-linked immunosorbent Assay (Ab-ELISA) as an epidemiological tool for the study of the prevalence of *T. vivax* in central Sudan**. *MSc thesis.* Khartoum: University of Khartoum; 2000.

11. Kheir SM, Abdel Alla HS, Rahman AHA: **A study on Tsetse and Tabanid Flies in Eastern Sudan**. *Sud J Vet Res* 1992, **11**:29-37.

12. Kheir SM, Abdel Alla HS, Rahman AHA: **Bovine Trypanosomiasis in Eastern Sudan**. *Sud J Vet Res* 1993, **12**:33-41.

13. Mahamed-Ahmed MM: **Distribution of Tsetse in Kurmuk District Blue Nile Provlnce/Sudan**. *Sud J Vet Se Anim Husb* 1989, **28**:45-54.

14. Mohamed-Ahmed M, Ahmed A, Ishag A: **Trypanosome infection rate of *Glossina morsitans submorsitans* in Bahr El Arab, South Darfur Province, Sudan**. *Trop Anim Health Pro* 1989, **21**(4):239-244.

15. Mohamed-Ahmed MM, Abdel Karim EI, Rahman AHA: **Field multiple drug-resistant bovine trypanosomes and recommendations for a new drug policy in South Darfur province, Sudan**. Khartoum: Veterinary Research Adminstration; 1988.

16. Mohamed-Ahmed MM, Abdel-Karim EI, Rahman AHA: **A successful Control of Multiple Drug Resistant Bovine Trypanosome Strains by Samorin Chemotherapy in South Darfur**. *Sud J Vet Res* 1992, **11**:15-27.

17. Nadia MO: **Molecular and parasitological survey on bovine trypanosomosis and sensitivity of *T. vivax* to isometamidium chloride**. Khartoum: Sudan Academy of Science; 2012.

18. Obaid BI: **Bovine Trypanosomosis and their Vectors in Bahr Alarab and Eldiain localities, South Darfur state**. *MSc thesis.* Khartoum: University of Khartoum; 2009.

19. Omer M, Ahmed A, Abusalab S: **A Retrospective Study on Animal Parasitic Diseases Diagnosed at Kassala Veterinary Research Lab (KVRL), Eastern Sudan**. *Vet Res* 2007, **1**(3):68-70.

20. Rahman A: **Observations on the epidemiology of bovine trypanosomosis in the Sudan**. Khartoum: University of Khartoum; 2002.

21. Rahman A, Elkhidir M: **A study on the ecology and trypanosome infection of *Glossina morsitans submorsitans* Newst in south Darfur, Sudan**. *Rev Elev Med Vet Pays Trop* 1984, **37**(Spécial):203-210.

22. Rahman AHA: **Observations on the trypanosomosis problem outside the tsetse belts of Sudan**. *Rev Sci Tech* 2005, **24**(3):965-972.

23. Rahman AHA, Goreish IA, Yagi RA, Rajab SA, Gasmir G: **The Effect of Bovine Trypanosomosis and Endo-Parasitism on Milk Production of a Dairy Farm in The White Nile State, Sudan**. *Sud J Vet Res* 2008, **23**:19-27.

24. Rahman AMA, Abdoon AMO, Elkhidir ME, Hall MJR: **A Tsetse and Trypanosomiasis Survey of South Kordofan Provinces, Sudan. I. Bovine trypanosomiasis**. *Sud J Vet Res* 1990-1991, **10**:1-12.

25. Rahman AMA, Abdoon AMO, Elkhidir ME, Hall MJR: **A Tsetse and Trypanosomiasis Survey of South Kordofan Provinces, Sudan. II. Entomological Aspects**. *Sud J Vet Res* 1990-1991, **10**:13-20.

26. Salim B, Bakheit MA, Salih SE, Kamau J, Nakamura I, Nakao R, Sugimoto C: **An outbreak of bovine trypanosomiasis in the Blue Nile State, Sudan**. *Parasit Vectors* 2011, **4**:74.

27. Osman OM, Musa MM: **The distribution and trypanosome infection rates of tsetse in Southern Darfur**. *The Sud J Vet Res* 1979, **1**:43-45.

28. Salih SE: **Studies on the epidemiology of bovine trypanosomosis in Blue Nile State, Sudan**. *MSc thesis.* Khartoum: Sudan Academy of Science; 2010.

29. Salma AR: **The pathogenicity and drug resistance of *Trypanosoma vivax* in calves and goats**. *PhD thesis.* Khartoum: University of Khartoum; 2005.

30. Sara MS: **Prevalence and risk factors of bovine trypanosomosis in Khartoum state, Sudan**. *MSc thesis.* Khartoum: Sudan University for Science and Technology; 2013.

31. Suliman TA: **Trypanosomiasis in Sinnar Area with some studies on related Diptera**. *MSc thesis.* Khartoum: University of Khartoum; 1992.

32. Yagi AI, Abdel Razig ME: **Eradication of *Glossina morsitans* (Diptera: Muscidae) in Koalib Hills, Nuba Mountains, Sudan**. *Sud J Vet Sci Anim Husb* 1969, **10**:33-54.

33. Veterinary Research Institute: **Annual report - Atbara**. Atbara: Veterinary Research Institute; 2006.

34. Veterinary Research Institute: **Annual report - Radom**. Radom: Veterinary Research Institute; 2006.

35. Veterinary Research Institute: **Annual report - Radom**. Radom: Veterinary Research Institute; 2007.

36. Veterinary Research Institute: **Annual report - Radom**. Radom: Veterinary Research Institute; 2008.

37. Veterinary Research Institute: **Annual report - Radom**. Radom: Veterinary Research Institute; 2009.

38. Veterinary Research Institute: **Annual report - Radom**. Radom: Veterinary Research Institute; 2010.

39. Veterinary Research Institute: **Annual report - Radom**. Radom: Veterinary Research Institute; 2011.

40. Veterinary Research Institute: **Annual report - Radom**. Radom: Veterinary Research Institute; 2012.

41. Veterinary Research Institute: **Annual report - Rabak**. Rabak: Veterinary Research Institute; 2006.

42. Veterinary Research Institute: **Annual report - Rabak**. Rabak: Veterinary Research Institute; 2007.

43. Veterinary Research Institute: **Annual report - Rabak**. Rabak: Veterinary Research Institute; 2008.

44. Veterinary Research Institute: **Annual report - Rabak**. Rabak: Veterinary Research Institute; 2009.

45. Veterinary Research Institute: **Annual report - Rabak**. Rabak: Veterinary Research Institute; 2010.

46. Veterinary Research Institute: **Annual report - Madani**. Madani: Veterinary Research Institute; 2007.

47. Veterinary Research Institute: **Survey of bovine trypanosomosis in the Blue Nile State**. Damazine: Veterinary Research Institute; 2011.

48. Veterinary Research Institute: **Annual report -Tsetse and Trypanosomosis Department**. Khartoum: Veterinary Research Institute; 1988.

49. Veterinary Research Institute: **Annual report -Tsetse and Trypanosomosis Department**. Khartoum: Veterinary Research Institute; 2005.

50. Veterinary Research Institute: **Annual report -Tsetse and Trypanosomosis Department**. Khartoum: Veterinary Research Institute; 2009.

51. Veterinary Research Institute: **Annual report -Tsetse and Trypanosomosis Department**. Khartoum: Veterinary Research Institute; 2010.

52. Veterinary Research Institute: **Annual report -Tsetse and Trypanosomosis Department**. Khartoum: Veterinary Research Institute; 2011.

53. Veterinary Research Institute: **Annual report -Tsetse and Trypanosomosis Department**. Khartoum: Veterinary Research Institute; 2012.

54. Ministry of Animal Resources: **Annual report - Gezeira state**. Ministry of Animal Resources; 2010.
